# A Vignetting Correction Method for Remote Sensing Images Based on Low-Rank Modeling and Polynomial Fitting

**DOI:** 10.3390/jimaging12070304

**Published:** 2026-07-07

**Authors:** Xue Zhao, Zhuoyue Hu, Zhengqin Xu

**Affiliations:** 1 State Key Laboratory of Infrared Physics, Shanghai Institute of Technical Physics, Chinese Academy of Sciences, Shanghai 200083, China; zhaoxue22@mails.ucas.ac.cn; 2 University of Chinese Academy of Sciences, Beijing 100049, China

**Keywords:** vignetting correction, low-rank modeling, polynomial fitting, spatial nonuniformity, optical vignetting

## Abstract

Vignetting introduces spatial radiometric nonuniformity into remote sensing images and degrades subsequent radiometric analysis, image interpretation, and calibration-related applications. To address this problem, this paper proposes a vignetting correction method based on low-rank modeling and polynomial fitting. The method constructs a multi-frame data matrix in the logarithmic domain, extracts the shared vignette component through rank-1 low-rank modeling, and further recovers a smooth vignette field through polynomial fitting. Experiments were conducted using real remote sensing images, simulated vignetted images, and star images. Among the three ablation variants, the proposed full method achieved the best performance, with MAE, MAD, Center-MAE, and Edge-MAE values of 0.48%, 3.65%, 0.14%, and 0.52%, respectively. Compared with the low-rank-only method, these metrics were reduced by 23.8%, 32.8%, 71.4%, and 20.0%, respectively. An additional all-frame comparison across 28 dataset settings showed that the proposed rank-1 model achieved mean accuracy comparable to nuclear-norm-based standard RPCA, while exhibiting lower cross-dataset variability in MAE, MAD, and Edge-MAE. For star images, the method reduced image-plane nonuniformity from 1.39–1.92% to 0.59–0.80% while preserving background-subtracted stellar DN values. These results demonstrate that the proposed method provides physically interpretable and stable vignetting correction while maintaining radiometric consistency.

## 1. Introduction

Vignetting is a common radiometric nonuniformity in imaging systems, typically manifested as a gradual attenuation of image brightness from the center toward the edges. For remote sensing images, this spatial radiometric nonuniformity not only degrades visual quality but also affects radiometric calibration, land surface parameter retrieval, target detection, and consistency in multi-scene image mosaicking [1,2]. In wide-swath imaging, multi-detector mosaicking, and thermal infrared remote sensing, vignetting, striping, and response drift are often coupled, making image nonuniformity more pronounced [3,4]. However, in the absence of vignetting-free ground-truth images or dedicated calibration data, vignetting correction usually has to be performed under non-reference conditions. In such cases, existing methods can only rely on single-image statistical priors or multi-image shared constraints to estimate the vignette field, making them more susceptible to interference from scene content, texture structures, striping, and response drift, which in turn affects the stability of vignette recovery and correction accuracy. Therefore, developing a stable, generalizable, and remote-sensing-oriented vignetting correction method remains of clear research value.

Image vignetting correction methods can be roughly divided into three categories. The first category consists of methods based on physical calibration or radiometric calibration, which recover the vignette distribution through exposure modeling, camera response estimation, flat-field calibration, or on-orbit calibration [5,6]. Such methods usually have good physical interpretability, but they often require additional calibration data, stable imaging conditions, or rigorous experimental procedures, resulting in high deployment costs in practical remote sensing tasks. The second category comprises methods based on single-image statistical priors, such as texture segmentation, gradient distribution symmetry, or radial gradient constraints, to estimate the vignette field [7,8]. These methods reduce dependence on external calibration data, but their estimation results are easily disturbed by scene content when image content is complex, texture structures are strong, or imaging geometry is non-ideal. The third category extends radiometric nonuniformity correction to multi-view or multi-frame scenarios and restores radiometric consistency through multi-image information [9,10,11], rather than explicitly extracting the vignette field. Existing studies mainly focus on single-image vignette fitting, physical calibration, or relative radiometric correction, while explicit modeling of a vignette structure shared by multiple images remains relatively rare, especially in remote sensing scenarios.

In imaging, vignetting is essentially a low-frequency and smooth spatial radiometric field. Therefore, polynomial models and their variants have long been important tools for vignette modeling. Classical methods usually employ two-dimensional polynomials, radial polynomials, local polynomials, or deformable radial polynomials to approximate the vignette distribution [12,13,14]. These methods offer simple models, few parameters, easy implementation, and low computational cost, making them particularly suitable for describing smooth, low-frequency brightness attenuation fields. For vignetting in remote sensing images, polynomial fitting can effectively recover large-scale brightness variation trends and is therefore highly practical in engineering applications. Nevertheless, polynomial models also have clear limitations. First, fitting from a single image is easily affected by surface textures and radiometric structures. Second, low-order polynomials are difficult to accurately describe complex off-center and asymmetric vignetting. Third, when multi-frame shared constraints are absent, the fitting result often exhibits large instability. In other words, polynomial fitting is effective in representing smooth low-frequency structures, but it is not well suited to separating the vignette field from complex image content.

Statistical analysis of long-term experimental data from space optical systems shows that, when the space environment and observation angle vary within a limited range, the vignette field usually changes slowly over a short time scale and is unlikely to undergo significant variations. Its spatial distribution and attenuation amplitude generally exhibit good stability. Therefore, it can be approximately regarded as satisfying the time window of stability assumption.

The worst-corner vignette degree is used for quantitative evaluation.

(1) $$V_{corner,worst}=1-\eta_{corner,min}$$

where $\eta_{corner,min}=\min\left(\frac{I_{LT}}{I_{c}},\frac{I_{RT}}{I_{c}},\frac{I_{LB}}{I_{c}},\frac{I_{RB}}{I_{c}}\right)$, $I_{c}={median}_{\left(x,y\right)\in \Omega_{c}}\hat{I}\left(x,y\right)$, $I_{LT}={median}_{\left(x,y\right)\in \Omega_{LT}}\hat{I}\left(x,y\right)$, $I_{RT}={median}_{\left(x,y\right)\in \Omega_{RT}}\hat{I}\left(x,y\right)$, $I_{LB}={median}_{\left(x,y\right)\in \Omega_{LB}}\hat{I}\left(x,y\right)$, $I_{RB}={median}_{\left(x,y\right)\in \Omega_{RB}}\hat{I}\left(x,y\right)$.

Here, $I_{c}$ denotes the median low-frequency radiance of the central region, $I_{LT}$ denotes the median low-frequency radiance of the upper-left region, $I_{RT}$ denotes the median low-frequency radiance of the upper-right region, $I_{LB}$ denotes the median low-frequency radiance of the lower-left region, $I_{RB}$ denotes the median low-frequency radiance of the lower-right region. $\hat{I}\left(x,y\right)$ denotes the low-frequency radiance field obtained by Gaussian low-pass filtering.

**Figure 1:**
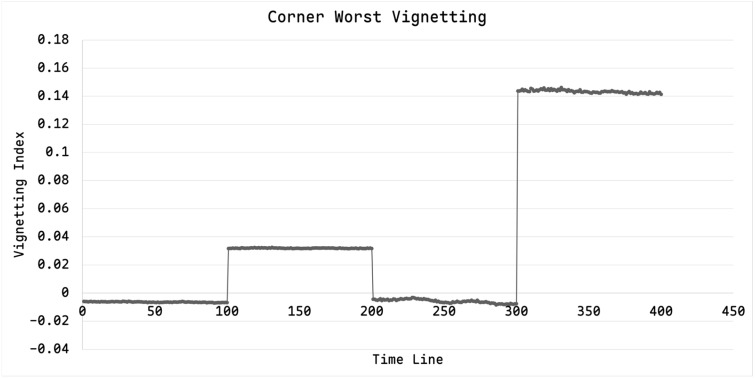
Temporal Invariance of Vignetting.

Figure 1 shows the variation of the estimated vignette degree obtained from multi-frame radiometric images acquired under different imaging conditions. The data used in this figure were derived from the star image sequence described in the dataset section. The low-frequency illumination distribution was extracted from each image, and the corresponding vignette degree was calculated to analyze the temporal stability of the vignette field. The vignette field usually satisfies the time window of stability assumption under similar observation directions and spatiotemporal imaging conditions; that is, its spatial distribution and attenuation characteristics remain approximately unchanged within a short time window. However, when the spatiotemporal observation environment changes significantly, the vignette field may become nonstationary, and its attenuation degree and spatial morphology may vary markedly or even exhibit abrupt changes. It should also be noted that, from the perspectives of radiometric field transmission and sensor response mechanisms, vignetting is mainly a multiplicative spatial attenuation process, whereas additive terms usually only represent background bias, response drift, or local additional disturbances. Based on this understanding, this paper models the vignette field as a multiplicative-dominant common component and treats additive effects as secondary disturbances.

The time window of stability used in this study should be understood as an operational stability interval rather than a fixed number of frames or a universal physical duration. It denotes a continuous acquisition period in which the observation geometry, instrument state, thermal condition, and acquisition configuration remain approximately unchanged, so that the vignette field can be regarded as quasi-stable. Vignetting Index is relatively stable within each acquisition segment, while step-like changes occur between different segments. These abrupt changes indicate that the imaging condition may have changed, and therefore images across different segments should not be jointly used to estimate a single shared vignette field.

Studies on low-rank decomposition have shown that, when an observation matrix can be decomposed into a low-rank background and sparse disturbances, stable recovery can be achieved through convex optimization or approximate decomposition [15,16,17]. This idea has since been widely used in background modeling, shadow correction, illumination field separation, and hyperspectral anomaly detection [18,19,20]. For vignette correction, if multiple frames share similar vignette distributions, while different scene contents, local anomalies, and noise mainly appear as non-shared disturbances, then organizing multi-frame data into a matrix and applying low-rank decomposition may make it possible to extract the common vignette structure. However, low-rank decomposition itself is more focused on structure extraction and does not directly guarantee that the recovered result satisfies the physical property of vignetting as a smooth low-frequency field. Therefore, using low-rank decomposition alone often fails to obtain an ideal vignette field. Modeling and solving the shared vignette field across multiple frames under the same imaging condition remain relatively uncommon, especially in remote sensing scenarios. Meanwhile, existing studies typically emphasize parametric fitting, with limited systematic analysis of the respective roles, complementarity, and joint influence of low-rank modeling and polynomial fitting on final vignette estimation and correction performance.

In addition to affecting ground radiometric consistency, vignetting also directly influences the accuracy of star-based radiometric calibration. Stars have been used as stable calibration sources for on-orbit radiometric calibration and imaging performance assessment of remote sensing satellites, and a key prerequisite is that the same star should exhibit a consistent radiometric response under different observation conditions and at different field positions [21,22]. However, vignetting introduces field-dependent multiplicative attenuation across the image plane, causing the point-spread energy distribution and the background-subtracted DN value of a star to vary with field position. This reduces the consistency of stellar signal extraction and further affects the stability of calibration results [23,24]. For low-irradiance stellar targets in particular, background subtraction and signal-to-noise control are critical to photometric accuracy. If vignetting is not effectively suppressed, the field-dependent response variation it introduces will be directly translated into calibration error. Existing studies have shown that star-based calibration can achieve approximately 2% calibration accuracy under reasonable signal-to-noise conditions [22], which implies that vignette correction should minimize field-dependent variations in stellar energy as much as possible to improve star-based calibration accuracy.

Because it is difficult to acquire strictly paired remote sensing images with and without the same vignette degradation under identical imaging conditions, simulation-based evaluation is often necessary for quantitative assessment. Similar strategies have been widely used in image restoration and enhancement studies. For example, Bal and Palus [25] used numerical simulations with known vignette functions to evaluate vignetting models, where the true degradation field is available for MAE and RMSE calculation. In the dehazing field, the RESIDE benchmark constructed large-scale synthetic hazy images from clean images using a physical atmospheric scattering model and combined full-reference, no-reference, and subjective evaluation criteria. Sakaridis et al. [26,27,28] also generated synthetic fog on real clear-weather images to build Foggy Cityscapes for evaluating vision algorithms under degraded imaging conditions. These studies indicate that applying a physically meaningful degradation model to real images is an accepted experimental strategy when paired real degraded/reference data are difficult to obtain.

This paper formulates remote sensing image vignette correction as a vignette-field extraction problem. Specifically, a multi-frame data matrix is first constructed in the logarithmic domain, and low-rank decomposition is used to estimate the common vignette field. Polynomial fitting is then introduced to further recover a smooth vignette field, thereby improving the physical plausibility and stability of the result. Finally, a correction gain is constructed in the original intensity domain to achieve image vignette correction. The proposed method accounts for both vignette-field extraction capability and low-frequency field modeling capability.

The main contributions of this paper are as follows.

(1) A multi-frame vignetting correction framework is proposed based on the short-term stability of the vignette field. Different from single-image prior-based methods, such as partial-gradient correction, and parameterized radial polynomial methods, the proposed framework exploits the shared vignette component among multiple frames. This design reduces the influence of scene-dependent textures and local radiometric variations and improves the robustness of vignette-field estimation.

(2) A correction strategy combining rank-1 shared low-rank modeling and polynomial fitting is developed. The rank-sensitivity experiment shows that the rank-1 setting achieves lower MAE, MAD, Center-MAE, and Edge-MAE than higher-rank diagnostic settings, supporting the use of a shared rank-1 vignette model. In addition, polynomial-order sensitivity analysis shows that the selected polynomial order provides a practical balance between fitting accuracy, spatial stability, and computational cost.

(3) Extensive experiments, including ablation studies, external baseline comparisons, stellar-image validation, convergence and runtime analysis, and parameter-sensitivity tests, demonstrate that the proposed method provides higher correction accuracy and better stability than the compared methods under the same evaluation metrics.

## 2. Materials and Methods

Based on the systematic analysis in the previous section, a remote sensing image vignette correction method based on low-rank modeling and polynomial fitting is proposed. The overall method jointly considers vignette-field extraction capability and smooth-field modeling capability.

To make the algorithm self-contained and easier to follow, the main variables used are summarized in Table 1.

Algorithm 1 summarizes the overall workflow of the proposed vignetting correction framework and its two ablation variants. The common preprocessing stage first transforms the input multi-frame vignetted images into the logarithmic domain and constructs a shared data matrix for subsequent estimation. The low-rank-only branch evaluates the effect of using only the shared low-rank component to recover the vignette field, while the polynomial-only branch evaluates the effect of relying only on shared image statistics and low-frequency polynomial fitting. The full method combines these two steps: the shared low-rank model first extracts the common vignette-related component from multiple frames, and polynomial fitting then refines this component into a smooth low-frequency vignette field. Finally, the estimated multiplicative vignette field is converted into a correction gain and applied to the input image to obtain the corrected output.
**Algorithm 1.** Proposed muli-frame vignetting correction method**Input**: Vignetted images $X={\left\{I_{k}\right\}}_{k=1}^{N}$, polynomial order P, and numerical constant ϵ.**Output**: Estimated vignette field $\hat{V}$ and corrected images ${\left\{I_{corr,k}\right\}}_{k=1}^{N}$.1: Transform each image into the logarithmic domain: $D_{k}=log\left(I_{k}+\epsilon \right)$.2: Vectorize $D_{k}$ and construct $D=\left[d_{1},\ldots ,d_{N}\right]$.3: Estimate the shared low-rank component by solving D=L+S, where $L=v1^{T}$.4: Reshape v to obtain the initial vignette field $B_{0}\left(x,y\right)$.5: Smooth $B_{0}$ and fit a polynomial of order P to obtain $\hat{B}\left(x,y\right)$.6: Recover the multiplicative vignette field: $\hat{V}\left(x,y\right)=exp\left(\hat{B}\left(x,y\right)\right)$.7: Calculate the correction gain $G\left(x,y\right)=1/\left(\hat{V}\left(x,y\right)+\epsilon \right)$.8: Correct each frame: $I_{corr,k}=clip\left(I_{k}G,0,L_{max}\right)$.9: Return $\hat{V}$ and ${\left\{I_{corr,k}\right\}}_{k=1}^{N}$.

### 2.1. Datasets

The experimental data used in this study consist of three parts: real remote sensing images, simulated vignetted images, and star images. These three types of data serve different purposes in the evaluation. The real remote sensing images are used to demonstrate the applicability of the proposed method under practical imaging conditions, the simulated vignetted images are used for controlled full-reference quantitative analysis, and the star images are used to further assess the influence of vignette correction on radiometric consistency and calibration-related analysis.

The real remote sensing images were collected from SDGSAT-1, Landsat 8/9, and ASTER, covering different imaging conditions and representative surface scenes. These data provide diverse image content and radiometric characteristics for evaluating correction performance under practical conditions. For full-reference quantitative evaluation, simulated vignetted images were generated by multiplicatively imposing vignette fields derived from independent detector radiometric images onto remote sensing radiance images. This semi-synthetic strategy preserves realistic scene textures and radiometric structures while introducing physically meaningful vignette degradation from measured detector data. The detector radiometric images used to prepare the imposed vignette fields were acquired separately from the remote sensing test images and were not included in the correction input sequence. During evaluation, the proposed method estimates the vignette field only from the simulated vignetted images, while the original remote sensing radiance images are used solely as reference images for calculating full-reference metrics. Therefore, the simulation procedure is independent of the correction process and does not introduce circular evaluation.

The remote sensing images and the simulated vignetted images were all of size 2048 × 2048 pixels, whereas the star images were of size 1024 × 1024 pixels. The star image dataset contains multiple stellar samples acquired under the experimental setting, and Table 2 shown in the manuscript are representative examples selected for visual comparison and quantitative illustration rather than the entire star dataset. As listed in Table 1, the star dataset is accompanied by auxiliary information such as stellar magnitude, right ascension, and declination, which is used to support star identification and subsequent calibration-related analysis.

### 2.2. Correction Method

The proposed correction method consists of four main stages. First, the multiplicative vignetting model is transformed into an additive representation in the logarithmic domain. Second, multi-frame images are organized into a shared data matrix. Third, the common vignette component is separated from non-shared scene variations using low-rank–sparse modeling. Finally, polynomial fitting is applied to recover a smooth vignette field, which is then converted into a correction gain in the original intensity domain.

#### 2.2.1. Logarithmic Domain Transformation

Considering that vignetting usually acts on an image as a multiplicative factor in the imaging model, the original image *I* can be expressed as:

(2) I=R·V+n

where R is the ideal image, V is the spatial vignette field, and n denotes the low-amplitude background noise.

Taking the logarithm of both sides yields the following approximate representation.

(3) logI⇔logR+logV

The logarithmic approximation is valid when the additive noise is much smaller than the multiplicative signal component, i.e., |n(x,y)| << |V(x,y)R(x,y)|. In this case, the noise-induced logarithmic term can be treated as a small residual perturbation. For low-SNR regions, this approximation may be less accurate; therefore, the resulting residual components are further mitigated through multi-frame shared modeling, sparse residual separation, and low-frequency polynomial fitting.

#### 2.2.2. Data Matrix Construction

Assume that there are n images in total, and that each image has a size of H×W=m. Each image is vectorized column-wise into a column vector of length, and all images are concatenated to obtain the observation matrix $D_{0}$.

Since the gray-value range of the original images is large, the matrix is first normalized as D to reduce dimensional effects and improve numerical stability.

#### 2.2.3. Vignetting Field-Sparse Anomaly Separation for Multi-Frame Images

This paper models the data matrix using a low-rank-sparse decomposition model, i.e.,

(4) D=L+S

where L represents the vignette field shared by multiple images, and S represents the non-shared residual term relative to the vignette field, including differences in scene content, local textures, noise, and disturbance terms.

The reason for modeling L as a low-rank term is that, under the time window of stability assumption of the vignette field, different images share the same or approximately the same vignette distribution, and their vectorized columns are therefore strongly correlated. In the ideal case, the shared vignette component can be represented as $L=v1^{T}$, corresponding to a rank-1 structure. It can therefore be approximately regarded as low-rank in practice. On the other hand, scene content and local disturbances appear as non-shared variations relative to the shared vignette component and can be further approximated as a sparse residual term S in the low-rank-sparse decomposition framework, where $v\in R^{m\times 1}$ denotes the estimated common background vector, and $1\in R^{m\times n}$ denotes an all-ones vector. This form means that each column of matrix L is identical; that is, all images share the same vignette field. Therefore,

(5) $$\underset{v,s}{\min}\;\lambda {\left\|S\right\|}_{1}\;\;\;s.t.D=v1^{T}+S$$

To solve the above problem, the Augmented Lagrangian Method (ALM) is adopted for iterative optimization, and the augmented Lagrangian function is constructed as:

(6) $$L\left(v,S,Y,\mu \right)=\lambda {\left\|S\right\|}_{1}+\langle Y,D-v1^{T}-S\rangle +\frac{\mu }{2}{\left\|D-v1^{T}-S\right\|}_{F}^{2}$$

where Y is the Lagrange multiplier, μ>0 is the penalty parameter, 〈·,·〉 denotes the matrix inner product, and ${\left\|\cdot \right\|}_{F}$ denotes the Frobenius norm and where λ is the regularization parameter of the sparse term. In this paper, $\lambda =\frac{1}{\sqrt{m}}$ is used to balance the vignette field and image textures.

During initialization, we first set:

(7) $$Y_{0}=D$$

Then, it is normalized using the joint dual norm,

(8) $$Y_{0}\leftarrow \frac{D}{max\left({\left\|D\right\|}_{2},{\left\|D\right\|}_{\infty }/\lambda \right)}$$

where ${\left\|D\right\|}_{2}$ denotes the spectral norm of the matrix, and ${\left\|D\right\|}_{\infty }=\underset{i,j}{\max}\left|D_{ij}\right|$ denotes the maximum absolute value among all matrix elements. This initialization keeps the multiplier variable within an appropriate numerical scale and improves the stability of subsequent iterations. The penalty parameter is initialized as:

(9) $$\mu_{0}=\frac{1.25}{{\left\|D\right\|}_{2}}$$

and its upper bound is set as:

(10) $$\mu_{max}=10^{7}\mu_{0}$$

At the *k*-th iteration, $S^{k}$, $Y^{k}$ and $\mu^{k}$ are first fixed, and the background vector v is updated. Define the intermediate variable,

(11) $${\tilde{L}}^{k}=D-S^{k}+\frac{1}{\mu^{k}}Y^{k}$$

Since the low-rank term is constrained to a column-shared structure $v1^{T}$, the update of v can be written as the following least-squares problem.

(12) $$v^{k+1}=arg\;\underset{v}{\min}{\left\|{\tilde{L}}^{k}-v1^{T}\right\|}_{F}^{2}$$

The closed-form solution ${\tilde{L}}^{k}$ is the row-wise mean along the column direction, i.e.,

(13) $$v_{i}^{k+1}=\frac{1}{n}\sum_{j=1}^{n}{\tilde{L}}_{ij}^{k},i=1,2,\ldots ,m$$

The low-rank term is then constructed as:

(14) $$L^{k+1}=v^{k+1}1^{T}$$

After obtaining $L^{k+1}$, fix $L^{k+1}$, $Y^{k}$, and $\mu^{k}$, and update the sparse term S. Define:

(15) $${\tilde{S}}^{k}=D-L^{k+1}+\frac{1}{\mu^{k}}Y^{k}$$

The subproblem of S can be written as:

(16) $$S^{k+1}=arg\;\underset{S}{\min}\;\lambda {\left\|S\right\|}_{1}+\frac{\mu^{k}}{2}{\left\|S-{\tilde{S}}^{k}\right\|}_{F}^{2}$$

Its closed-form solution is given by the soft-thresholding operator,

(17) $$S^{k+1}=S_{\lambda /\mu^{k}}\left({\tilde{S}}^{k}\right)$$

where

(18) $$S_{\tau }\left(x\right)=sign\left(x\right)max\left(\left|x\right|-\tau ,0\right)$$

The reconstruction residual at the current iteration is computed as:

(19) $$Z^{k+1}=D-L^{k+1}-S^{k+1}$$

and the Lagrange multiplier is updated as:

(20) $$Y^{k+1}=Y^{k}+\mu^{k}Z^{k+1}$$

The penalty parameter is increased according to:

(21) $$\mu^{k+1}=min\left(\rho \mu^{k},\mu_{max}\right)$$

where ρ=1.1. Compared with a larger growth factor, this setting avoids excessively rapid growth of the penalty parameter, which may otherwise cause the iterations to be prematurely locked and thus hinder sufficient convergence of the background term.

To determine whether the algorithm has converged, the normalized residual is defined as:

(22) $${Err}^{k+1}=\frac{{\left\|Z^{k+1}\right\|}_{F}}{{\left\|D\right\|}_{F}}$$

To monitor the stability of the common background vector v, its relative change rate is further computed as:

(23) $$∆v^{k+1}=\frac{{\left\|v^{k+1}-v^{k}\right\|}_{2}}{{\left\|v^{k}\right\|}_{2}+10^{-6}}$$

Based on the above two criteria, the iteration is terminated when ${Err}^{k+1}\leq 10^{-5}$ and $∆v^{k+1}\leq 10^{-4}$ are simultaneously satisfied. In addition, the maximum number of iterations is set to 200. If this limit is reached before both conditions are satisfied, the iteration is terminated and recorded as reaching the maximum iteration limit.

Although low-rank decomposition can extract the common-structure vignette field from multiple images, the constraint in (5) does not impose smoothness. For vignetting, the true vignette field usually appears as a continuous, low-frequency, and smoothly varying spatial radiometric attenuation. The result obtained by low-rank decomposition alone may still contain local fluctuations, residual textures, or non-ideal structures. Therefore, polynomial fitting is further introduced after low-rank decomposition to obtain a vignette field that better matches practical imaging conditions.

#### 2.2.4. Two-Dimensional High-Order Polynomial Fitting

We define the obtained vignette mask as l, and subtract the global mean of the logarithmic observation matrix to remove the overall bias,

(24) $$l_{adj}=l-\bar{D_{log}}$$

It is then reshaped into a two-dimensional image, defined as $\Gamma \in R^{H\times W}$, which serves as the initial estimate of the two-dimensional vignette field.

Since the directly decomposed result Γ may still contain local undulations and numerical fluctuations, a two-dimensional high-order polynomial fitting is further performed to obtain a smooth, continuous, and analytically representable vignette field.

Grid coordinates are constructed and normalized, with the coordinates set as: x=1,2,…,W, y=1,2,…,H

(25) $$X^{\prime }=\frac{X-\bar{X}}{W/2},\;Y^{\prime }=\frac{Y-\bar{Y}}{H/2}$$

where $\bar{X}$ and $\overset{¯}{Y}$ are the means of the horizontal and vertical coordinates, respectively.

The fitted surface is defined as:

(26) $$P\left(X^{\prime },Y^{\prime }\right)=\sum_{p+q\leq P}a_{pq}{\left(X^{\prime }\right)}^{p}{\left(Y^{\prime }\right)}^{q}$$

where $a_{pq}$ denotes the polynomial coefficient to be solved, the indices satisfy p,q≥0, and P is the polynomial order.

The total number of coefficients in the two-dimensional P polynomial is:

(27) $$Q=\frac{\left(P+1)(P+2\right)}{2}$$

The two-dimensional image Γ is vectorized column-wise into a column vector.

(28) $$z=vec\left(\Gamma \right)\in R^{m\times 1}$$

All basis functions ${\left(X^{\prime }\right)}^{p}{\left(Y^{\prime }\right)}^{q}$ are expanded term by term and concatenated to construct the design matrix.

(29) $$A=\left[\begin{array}{ccccccc} 1 & X_{1}^{\prime } & Y_{1}^{\prime } & {\left(X_{1}^{\prime }\right)}^{2} & X_{1}^{\prime }Y_{1}^{\prime } & {\left(Y_{1}^{\prime }\right)}^{2} & \ldots \\ 1 & X_{2}^{\prime } & Y_{2}^{\prime } & {\left(X_{2}^{\prime }\right)}^{2} & X_{2}^{\prime }Y_{2}^{\prime } & {\left(Y_{2}^{\prime }\right)}^{2} & \ldots \\ \vdots & \vdots & \vdots & \vdots & \vdots & \vdots & \ddots \\ 1 & X_{m}^{\prime } & Y_{m}^{\prime } & {\left(X_{m}^{\prime }\right)}^{2} & X_{m}^{\prime }Y_{m}^{\prime } & {\left(Y_{m}^{\prime }\right)}^{2} & \ldots \end{array}\right]$$

The coefficient vector is denoted by:

(30) $$p={\left[a_{00},a_{10},a_{01},a_{20},a_{11},a_{02},\ldots \right]}^{T}$$

The fitting model can then be written as:

(31) Ap≈z

The coefficients are solved by least squares:

(32) $$p=\arg\underset{p}{\min}{\left\|Ap-z\right\|}_{2}^{2}$$

After fitting, the smooth surface $\Gamma_{smooth}$ is reconstructed.

Finally, the minimum value is subtracted so that the vignette field is zero-referenced, yielding the final smooth vignette field.

(33) $$\Gamma_{final}=\Gamma_{smooth}-min\left(\Gamma_{smooth}\right)$$

In summary, this section formulates the proposed vignetting correction framework from both the physical imaging assumption and the algorithmic implementation. The logarithmic transformation converts the multiplicative vignette degradation into an additive form, enabling the shared vignette component to be estimated through low-rank modeling. Polynomial fitting is then introduced to enforce the smooth low-frequency property of the vignette field, and the corrected image is finally recovered in the original intensity domain. The defined evaluation metrics further provide a quantitative basis for assessing global correction accuracy, spatial uniformity, and regional error distribution. Based on this formulation, the experimental validation and comparative analysis are presented in the following section.

## 3. Results

This section evaluates the proposed method from both quantitative and qualitative perspectives. The evaluation metrics and their applicable data types are first introduced. The recovered vignette fields, corrected remote sensing images, statistical comparisons, and star image results are then analyzed to assess correction accuracy, spatial uniformity, radiometric consistency, and the respective contributions of low-rank modeling and polynomial fitting.

### 3.1. Quantitative Evaluation

For quantitative analysis of the final extracted results, four metrics are used: mean absolute error (MAE), maximum absolute deviation (MAD), center-region mean absolute error (Center-MAE), and edge-region mean absolute error (Edge-MAE).

Let the corrected image be $\hat{I}$, the vignette-free reference image be $I_{gt}$, and the image size be  H×W. Denote the total number of pixels by N=H×W. The pixel-wise error is defined as:

(34) $$e\left(i,j\right)=\hat{I}\left(i,j\right)-I_{gt}\left(i,j\right)$$

Thus, MAE, MAD, Center-MAE, and Edge-MAE are expressed as follows.

(35) $$MAE=\frac{\frac{1}{N}\sum_{i=1}^{H}\sum_{j=1}^{W}\left|e\left(i,j\right)\right|}{L}$$

(36) $$MAD=\frac{\underset{i,j}{\max}\left|e\left(i,j\right)\right|}{L}$$

where L is the upper limit of the dynamic range; it is 4095 for 12-bit images and 65,535 for 16-bit images.

(37) $$CerterMAE=\frac{\left(\frac{1}{\left|\Omega_{c}\right|}\sum_{\left(i,j\right)\in \Omega_{c}}\left|e\left(i,j\right)\right|\right)}{L}$$

(38) $$EdgeMAE=\frac{\left(\frac{1}{\left|\Omega_{e}\right|}\sum_{\left(i,j\right)\in \Omega_{e}}\left|e\left(i,j\right)\right|\right)}{L}$$

where $r\left(i,j\right)=\sqrt{{\left(\frac{j-x_{c}}{W/2}\right)}^{2}+{\left(\frac{i-y_{c}}{H/2}\right)}^{2}}\;$ denotes the normalized radial distance, the center region is $\Omega_{c}=\left\{\left(i,j\right)\;|\;r\left(i,j\right)\leq 0.3\right\}$, and the edge region is $\Omega_{e}=\left\{\left(i,j\right)\;|\;0.3\leq r\left(i,j\right)\leq 1.0\right\}$.

For star images, the quantitative evaluation in this study is conducted from two aspects. First, the direct purpose of vignette correction is to reduce spatial nonuniformity in image-plane radiometric response. If the consistency of stellar responses across the image plane is improved after correction, then the spatial nonuniformity caused by vignetting can be considered effectively suppressed. The image-plane nonuniformity metric is therefore used for this purpose.

(39) $$UR=\frac{1}{\bar{R}}\sqrt{\frac{1}{H\times W}\sum_{i=1}^{H}\sum_{j=1}^{W}{\left[R\left(i,j\right)-\bar{R}\right]}^{2}}\times 100\%$$

Second, vignette correction is intended to restore response uniformity rather than alter the intrinsic radiometric information of the target itself. Therefore, after local background subtraction, the net stellar DN value should remain as stable as possible. If the correction process significantly changes the background-subtracted stellar net signal, it indicates that additional radiometric bias has been introduced while removing vignetting. Based on these considerations, the correction performance of different methods is quantitatively evaluated in terms of both nonuniformity reduction and preservation of net stellar DN values.

(40) $$D_{net}=D_{star}-{\bar{D}}_{bg}$$

where $D_{net}$ denotes the net stellar DN value, $D_{star}$ denotes the DN response of the target region, and ${\bar{D}}_{bg}$ denotes the DN value of the local background region.

### 3.2. Analysis of Ablation Results

This section further analyzes the role of polynomial fitting after low-rank decomposition and provides a quantitative comparison of different correction strategies. The purpose is to clarify why low-rank decomposition alone is insufficient to recover a physically plausible vignette field, and how polynomial fitting improves the spatial smoothness and low-frequency consistency of the estimated field. In addition to the ablation comparison among the low-rank-only, polynomial-only, and full methods, representative external baseline methods are also included to evaluate the competitiveness of the proposed method under the same datasets and metrics.

**Figure 2:**
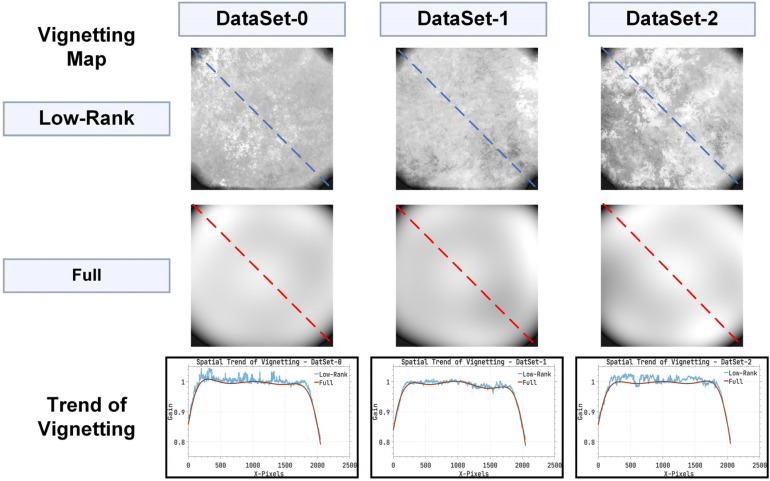
Necessity of Polynomial Fitting.

As shown in Figure 2, the vignette field obtained after low-rank decomposition mainly characterizes the common structural component in multiple images. However, the result may still be affected by local residual textures and non-smooth disturbances, and is therefore insufficient to fully represent the vignette distribution of the actual imaging system. After further polynomial fitting, the obtained vignette field better conforms to the physical characteristics of the true vignette field.

**Figure 3:**
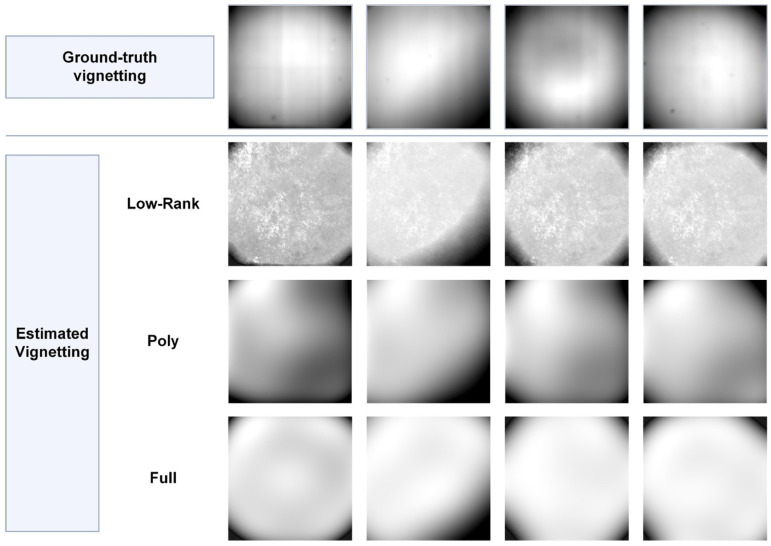
Extracted vignetting masks of different methods.

**Figure 4:**
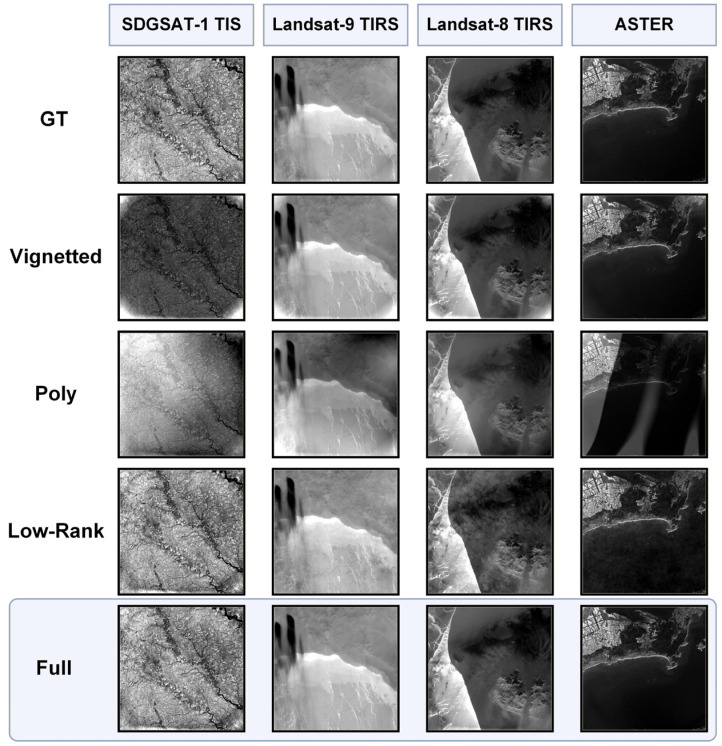
Correction results obtained by different methods.

As shown in Figure 3 and Figure 4, the vignette fields recovered by the three methods and their final correction results are further visualized. The correction performance of each method is analyzed and summarized based on the measured results.

**Figure 5:**
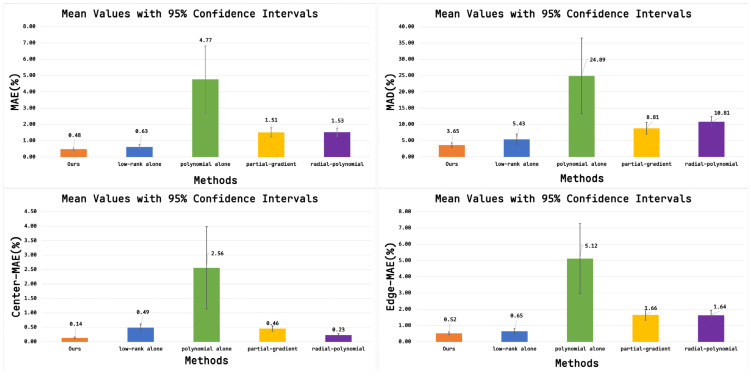
Quantitative evaluation.

For the four datasets, the results of the three methods in terms of MAE, MAD, Center-MAE, and Edge-MAE are shown in Figure 5. The proposed full method achieves the best overall performance among the three ablation variants, with MAE, MAD, Center-MAE, and Edge-MAE values of 0.48%, 3.65%, 0.14%, and 0.52%, respectively. Compared with the low-rank-only method, these four metrics are reduced by 23.8%, 32.8%, 71.4%, and 20.0%, respectively. Compared with the polynomial-only method, the reductions are 89.9%, 85.3%, 94.5%, and 89.8%, respectively. These results indicate that low-rank modeling can effectively extract the common vignette structure, while polynomial fitting further improves the smoothness and correction accuracy of vignette estimation.

To provide a more comprehensive comparison, two representative external baseline methods were included in addition to the ablation variants. The first baseline is a non-radial gradient-based method, which represents single-image prior-based correction strategies. This method estimates the low-frequency vignette field from the horizontal and vertical image gradients in the logarithmic domain and uses robust weighting to reduce the influence of strong scene edges. Since it does not impose radial symmetry, it can describe general two-dimensional low-frequency variations, but it remains sensitive to scene-dependent gradients.

The second baseline is a deformable polynomial fitting method, which represents parameterized radial or deformable vignetting models. It first estimates a smooth low-frequency field from a single vignetted image and then fits a deformable radial polynomial model with adjustable center and elliptical deformation. This strategy introduces stronger spatial smoothness and geometric constraints, but it lacks the multi-frame shared-structure constraint used in the proposed method.

All parameter values listed in Table 3 were fixed for all datasets without dataset-specific tuning. Ground-truth images were used only to calculate the evaluation metrics and were not involved in parameter selection or vignette-field estimation.

As shown in Table 4, the proposed method achieves consistently lower errors than the two external baseline methods across all four metrics. Specifically, the partial-gradient baseline obtains MAE, MAD, Center-MAE, and Edge-MAE values of 1.51%, 8.81%, 0.46%, and 1.66%, respectively, whereas the proposed method reduces them to 0.48%, 3.65%, 0.14%, and 0.52%. This corresponds to reductions of approximately 68.2%, 58.6%, 69.6%, and 68.7%, respectively. Compared with the radial-polynomial baseline, whose corresponding errors are 1.53%, 10.81%, 0.23%, and 1.64%, the proposed method reduces these four metrics by approximately 68.6%, 66.2%, 39.1%, and 68.3%, respectively.

These results indicate that single-image prior-based methods are less robust when scene-dependent low-frequency variations are mixed with the vignette component. The partial-gradient method is affected by image gradients caused by terrain textures or radiometric transitions, while the radial-polynomial method is constrained by its parametric radial assumption and may not fully describe non-ideal or asymmetric edge attenuation. In contrast, the proposed method first extracts the shared vignette-related component from multiple frames and then refines it through polynomial fitting, leading to better global accuracy and more stable correction in both central and edge regions.

To examine whether the explicit rank-1 model sacrifices performance relative to a general low-rank constraint, a nuclear-norm-based standard RPCA baseline [29,30] was additionally implemented. Standard RPCA estimates the low-rank and sparse components by solving

(41) $$\underset{L,S}{\min}{\left\|L\right\|}_{*}+\lambda {\left\|S\right\|}_{1}\;\;\;s.t.D=L+S$$

Here, ${\left\|L\right\|}_{*}$ denotes the nuclear norm, defined as the sum of the singular values of L, and ${\left\|S\right\|}_{1}$ promotes sparsity in the non-shared component. Unlike the proposed model $L=v1^{T}$, standard RPCA does not prescribe the rank of L. For fairness, both methods used all available frames and the same logarithmic transformation, polynomial post-fitting, gain construction, and evaluation metrics. Standard RPCA was performed at one-quarter linear spatial resolution and resized to the original resolution using bicubic interpolation.

As shown in Table 5, standard RPCA obtains slightly lower mean errors than the proposed method, but the absolute differences in MAE, MAD, Center-MAE, and Edge-MAE are only 0.02, 0.19, 0.02, and 0.01 percentage points, respectively. In contrast, the proposed method reduces the cross-dataset standard deviations of MAE, MAD, and Edge-MAE by approximately 29.2%, 14.1%, and 40.0%, respectively, while the Center-MAE standard deviation remains unchanged. These results indicate that the general low-rank model provides a small improvement in mean accuracy, whereas the explicit rank-1 model provides comparable accuracy, lower cross-dataset variability, and a more direct physical representation of the shared vignette field.

### 3.3. Qualitative Evaluation on Stellar Images

Stellar images provide an independent way to evaluate whether vignetting correction improves image-plane radiometric uniformity while preserving the radiometric response of point targets. In this section, the background-subtracted stellar DN values and image-plane nonuniformity are analyzed before and after correction. The results show that the proposed correction effectively reduces low-frequency background nonuniformity, whereas the net stellar DN values remain nearly unchanged. This suggests that the correction process mainly restores image-plane response consistency without introducing appreciable bias into the intrinsic radiometric signal of the stars.

**Figure 6:**
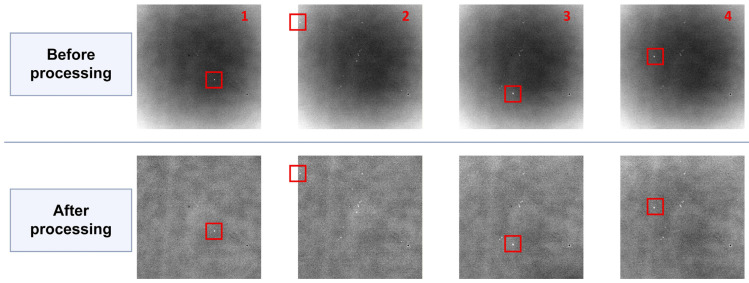
Star images before and after correction.

The visual results in Figure 6 further show the stellar images before and after vignetting correction. The red boxes mark the target-star regions that should be mainly focused on, while the surrounding background and the overall image-plane brightness distribution are also important for evaluating the correction effect. Before correction, obvious spatial brightness nonuniformity can be observed, and the background response varies across the image plane. After correction, the large-scale background nonuniformity is suppressed, and the response consistency around the stellar targets is improved. Meanwhile, the target stars marked by the red boxes remain clearly visible, indicating that the correction process mainly compensates for the low-frequency vignette field without significantly weakening the local stellar signal. Taken together, these results indicate that the proposed method reduces image-plane nonuniformity while preserving the stability of net stellar DN values, which is beneficial for subsequent star-based calibration analysis.

As shown in Table 6, the image-plane nonuniformity of the four stars samples are significantly reduced after vignette correction. Specifically, the nonuniformity metric decreases from 1.39%, 1.82%, 1.90%, and 1.92% before correction to 0.59%, 0.70%, 0.77%, and 0.80% after correction, respectively. This indicates that the response consistency at different field positions is substantially improved after correction. The result demonstrates that the proposed method can effectively suppress the spatial nonuniformity caused by vignetting and enhance the stability of stellar responses.

### 3.4. Convergence, Computational Cost, and Parameter Sensitivity

All experiments were implemented in MATLAB R2025a Update 1 and executed on a workstation equipped with an Intel Core i7-13700F CPU (16 cores and 24 logical processors) and 64 GB of RAM. The current implementation uses double-precision computation and does not explicitly employ GPU acceleration.

To evaluate the engineering feasibility of the proposed method, the computational cost was measured over 28 dataset settings using 100 frames of size 2048 × 2048 for vignette-field estimation.

For a 100-frame stack, a single double-precision matrix of size 2048×2048×100 requires approximately 3.13 GB. As shown in Table 7, because the current MATLAB implementation simultaneously maintains several matrices of this size, the core matrix storage is approximately 21.9 GB, and the peak memory requirement is estimated to be 22–30 GB when temporary arrays are included. This substantial memory requirement limits the current implementation primarily to offline ground-based processing on workstations rather than resource-constrained onboard or real-time deployment. It should be noted that this estimate reflects the present implementation rather than an irreducible theoretical cost of the proposed model. Future work will therefore investigate block-wise processing, frame batching, reduced-precision storage, and incremental low-rank updates to reduce memory consumption and improve deployment efficiency.

To further evaluate the practical applicability of the proposed method, this section analyzes the convergence behavior, computational cost, and sensitivity to the polynomial order. These factors are important for applying the method to high-resolution multi-frame images, especially for 2048 × 2048 remote sensing data.

In the ALM-based low-rank decomposition, convergence is monitored using two criteria: the normalized reconstruction residual and the relative change of the shared vignette vector. The normalized residual measures whether the constraint D=L+S is gradually satisfied, while the relative vector change reflects whether the estimated shared vignette field becomes stable. The iteration is terminated when both criteria are smaller than the predefined thresholds, or when the maximum number of iterations is reached. In this study, the residual threshold, vector-change threshold, and maximum number of iterations were set to $10^{-5},10^{-4}$, and 200, respectively.

**Figure 7:**
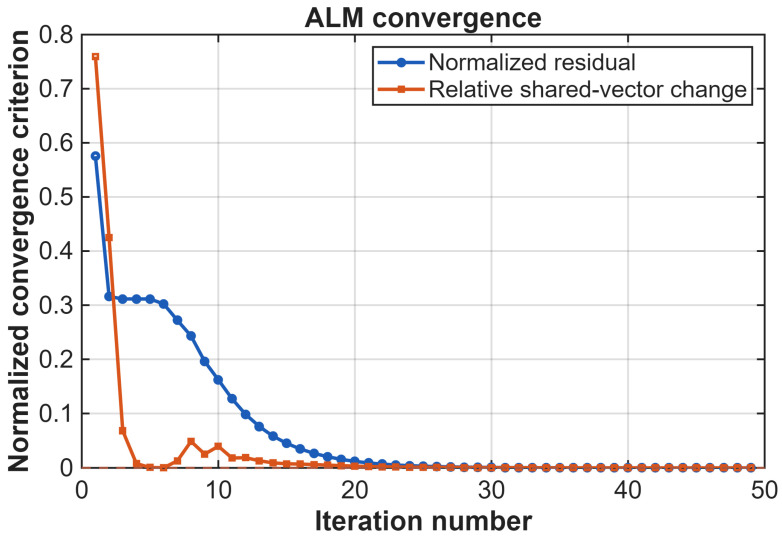
Convergence behavior of the ALM algorithm on DATA-3 using the full 176-frame stack. Both stopping criteria were satisfied after 49 iterations.

Figure 7 presents the convergence history for DATA-3, where both stopping criteria were satisfied after 49 iterations. As shown in Table 8, across the 28 test sequences derived from the four base datasets, the algorithm required 49.21 ± 0.42 iterations (mean ± SD), with a range of 49–50 iterations. All runs converged before reaching the maximum limit of 200 iterations, indicating consistent convergence behavior across the evaluated datasets. This indicates that the ALM-based decomposition reaches a stable numerical solution for the tested 2048 × 2048 multi-frame image stack.

Let M=H×W denote the number of pixels in each image, N the number of input frames, K the number of ALM iterations, and Q=(P+1)(P+2)/2 the number of polynomial basis terms. The logarithmic transformation and vectorization require O(MN) operations. In the ALM stage, each iteration updates the shared component, sparse component, residual, and Lagrange multiplier over the data matrix $D\in R^{M\times N}$, resulting in a per-iteration cost of O(MN). Therefore, the ALM decomposition has a total complexity of O(KMN). The polynomial fitting stage requires $O(MQ^{2}+Q^{3})$, and the final pixel-wise correction requires O(MN). Overall, the computational complexity is approximately $O(KMN+MQ^{2}+Q^{3})$, with a memory complexity of O(MN). Since P=6 in this study, Q=28, and the polynomial fitting cost is much smaller than the ALM decomposition cost for 2048 × 2048 multi-frame images.

The number of input frames determines the amount of redundant information available for separating the shared vignette field from scene-dependent variations. Insufficient frames may result in an unstable estimate because the diversity of scene content is inadequate to suppress non-shared structures. To evaluate this effect, the number of frames used for vignette estimation was set to $N_{f}=\{5,10,20,50,100,all\}$, and the results were compared with those obtained using all available frames.

As shown in Table 9, the results show that using only five frames leads to substantial performance degradation, indicating that insufficient scene diversity cannot reliably separate the shared vignette field from non-shared image content. Although the errors generally decrease as more frames are included, the trend is not strictly monotonic; the results at $N_{f}=20$ are slightly worse than those at $N_{f}=10$. This suggests that performance depends not only on the number of frames but also on the scene composition of the selected subset. When $N_{f}$ increases to 100, all four metrics are within approximately 10% of the all-frame results. Therefore, approximately 100 frames can be regarded as the empirical minimum for stable recovery under the tested conditions, rather than a universal theoretical threshold.

The polynomial order P is an important hyperparameter because it controls the balance between model flexibility and spatial smoothness. A low polynomial order may underfit complex vignette distributions, whereas an excessively high order may introduce unnecessary local fluctuations and reduce the physical smoothness of the estimated field. Therefore, a sensitivity analysis was conducted by comparing different polynomial orders under the same datasets and evaluation metrics. The results show that P=6 provides a good trade-off between correction accuracy and stability, and it was therefore used in all experiments.

As shown in Table 10, the polynomial order increases from P=2 to P=6, the correction errors decrease markedly, with MAE, MAD, and Edge-MAE reduced from 1.37%, 13.48%, and 1.80% to 0.42%, 3.72%, and 0.50%, respectively. This demonstrates that very low-order polynomials tend to underfit the vignette field. Further increasing the order to P=8 or P=10 does not provide consistent improvement: the MAD is slightly reduced at P=8, but the MAE and Center-MAE increase, suggesting potential local instability or overfitting. The runtime varies only slightly across different orders, indicating that the overall computational cost is dominated by the low-rank decomposition rather than the polynomial fitting. Therefore, P=6 was adopted as a balanced setting between fitting accuracy, spatial stability, and computational cost.

The regularization parameter λ controls the balance between the shared low-rank component and the sparse non-shared component. An inappropriate value may cause the shared vignette structure to leak into the sparse component or allow scene-dependent variations to remain in the estimated low-rank component. Therefore, the sensitivity to λ was evaluated on the representative DATA-0-3 dataset using all available frames. Specifically, λ was defined as $\lambda =\eta /\sqrt{m}$, where m=H×W and the scaling factor was set to η∈{0.25,0.5,1,2,4}. All other parameters were kept unchanged. The results are presented in Table 5.

As shown in Table 11, as the scaling factor η varied from 0.25 to 4, MAE and Edge-MAE remained unchanged at 0.22% and 0.24%, respectively. MAD varied only between 2.25% and 2.31%, while Center-MAE varied between 0.07% and 0.08%. These results indicate that the proposed method is insensitive to λ within the tested range. The default setting η=1 was selected because it achieved the lowest MAD and Center-MAE.

In the present implementation, the low-rank component is explicitly modeled as a rank-1 shared vignette field, $L=v1^{T}$, where v represents the common spatial vignette vector shared by all frames. Therefore, the rank used in the matrix decomposition is fixed to one in all main experiments. This choice is based on the short-term stability assumption of vignetting, under which multiple images acquired under similar imaging conditions are expected to share the same dominant vignette field.

Since the proposed method explicitly adopts a rank-1 shared vignette model, an additional rank-sensitivity experiment was conducted to examine the effect of rank selection. In this diagnostic experiment, the low-rank component was estimated with different fixed ranks, while the subsequent polynomial fitting and correction procedures were kept unchanged. As shown in Table 9, increasing the rank changes the balance between average correction accuracy and local error control. A higher rank may improve the representation flexibility of the low-rank component, but it may also absorb scene-dependent structures or local fluctuations, leading to increased worst-case errors. This result supports the use of the rank-1 shared model under the short-term stability assumption and also indicates that adaptive-rank modeling may be necessary for more complex cases.

To evaluate the influence of rank selection, a diagnostic rank-sensitivity experiment was conducted while keeping the subsequent polynomial fitting and correction procedures unchanged. As shown in Table 12, the rank-1 setting achieves the lowest errors, with MAE, MAD, Center-MAE, and Edge-MAE values of 0.20%, 1.14%, 0.07%, and 0.15%, respectively. When the rank is increased to 5, these errors increase to 0.29%, 1.57%, 0.10%, and 0.30%, respectively. Compared with rank 5, the rank-1 setting reduces MAE, MAD, Center-MAE, and Edge-MAE by approximately 31%, 27%, 30%, and 50%.

This result indicates that the dominant vignette component in the tested data is better represented by a rank-1 shared structure. Increasing the rank does not necessarily improve correction accuracy; instead, it may absorb scene-dependent variations or local residual structures into the low-rank component, reducing the physical stability of the estimated vignette field. Therefore, the rank-1 setting used in the proposed method is appropriate under the short-term stable vignetting assumption.

(42) $$V_{mix}=\sqrt{V_{A}V_{B}}$$

**Figure 8:**
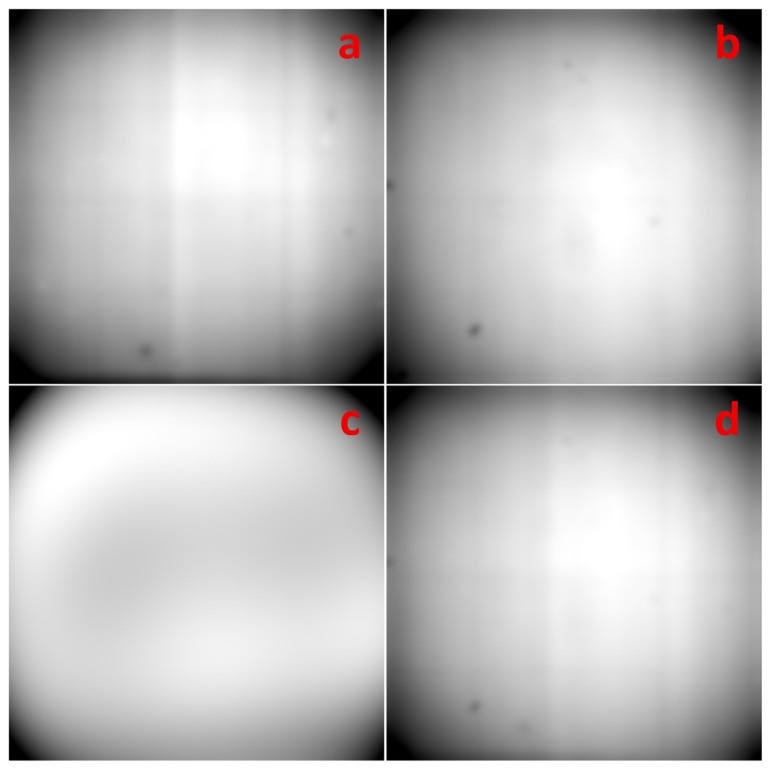
Failure-case analysis under non-shared vignette fields. (**a**,**b**) Two ground-truth vignette fields; (**c**) jointly estimated rank-1 vignette field; (**d**) theoretical compromise field.

The theoretical field $V_{mix}$ represents the idealized compromise solution of a single rank-1 shared model. It is not the theoretical output of standard RPCA, which permits multiple low-rank variation modes, as shown in Figure 8.

As shown in Table 13, applying a single jointly estimated rank-1 vignette field to the mixed sequence results in MAE values of 0.68% and 0.40% for Data A and Data B, respectively. This asymmetric residual error indicates that the jointly estimated field cannot accurately represent both vignette patterns and instead behaves as a compromise between them. After dividing the sequence into two stable windows and independently estimating the vignette field for each group, the MAE values decrease to 0.32% and 0.22%, corresponding to reductions of 52.9% and 45.0%, respectively. These results confirm that violation of the shared-field assumption leads to appreciable residual correction errors, whereas separate-window estimation effectively restores the validity of the rank-1 model and substantially improves correction accuracy.

Standard RPCA was additionally examined as a general low-rank reference. Because it does not enforce $L=v1^{T}$, it can preserve multiple low-rank variation modes when the sequence contains different vignette fields. However, standard RPCA does not automatically provide one vignette field for each temporal segment. If its low-rank columns are aggregated into a single field, the resulting estimate still cannot accurately represent both $V_{A}$ and $V_{B}$. Temporal windowing or clustering of the low-rank columns is therefore still required.

In practical applications, the vignetting index introduced above can provide a measurable basis for detecting changes in the vignette field. For a sequential image stack, the index can be calculated for each frame or short sliding window and smoothed to suppress random fluctuations. A candidate transition can then be detected by comparing the median vignetting indices of two adjacent windows. Specifically, a change point is declared when their difference exceeds a threshold determined from the index fluctuations observed under stable imaging conditions and persists for several consecutive frames. A minimum segment length can also be imposed to avoid excessive segmentation. After detection, the sequence is divided into separate stability windows, and the proposed rank-1 estimation is performed independently within each window. Nevertheless, automatic threshold selection may still be affected by scene-content changes and measurement noise. Therefore, developing and validating an adaptive change-point detector based on the vignetting index will be considered in future work.

## 4. Discussion

The results show that the proposed full method achieves the best overall performance, indicating that low-rank modeling and polynomial fitting contribute in complementary ways to vignette correction. Specifically, the full method yields MAE, MAD, Center-MAE, and Edge-MAE values of 0.48%, 3.65%, 0.14%, and 0.52%, respectively. Compared with the low-rank-only method, these four metrics are reduced by 22.9%, 32.9%, 71.7%, and 19.9%, respectively; compared with the polynomial-only method, the reductions are 89.9%, 85.4%, 94.6%, and 89.9%, respectively. These results indicate that low-rank modeling can effectively extract the common vignette structure, whereas polynomial fitting further improves the smoothness of vignette estimation and the final correction accuracy.

The comparison with standard RPCA further clarifies the trade-off introduced by the explicit rank-1 constraint. Standard RPCA provides slightly lower mean errors because its general low-rank component can represent multiple variation modes. However, the proposed method exhibits lower cross-dataset variability in MAE, MAD, and Edge-MAE. Therefore, the rank-1 model should not be interpreted as universally more accurate than standard RPCA; its advantages mainly lie in its direct physical interpretation and more stable cross-dataset behavior.

From a mechanistic perspective, low-rank modeling effectively extracts the vignette component shared by multiple images acquired under similar conditions, whereas polynomial fitting further enforces the smooth low-frequency property expected for a physically plausible vignette field. Therefore, the former mainly captures shared structure, while the latter refines its spatial representation.

Compared with the low-rank-only method, the results of the full method suggest that low-rank decomposition alone is not sufficient to recover a physically realistic vignette field. Although low-rank modeling can isolate the common component across multiple images, the estimated field may still contain local fluctuations and non-ideal structures. These residual components are not fully consistent with the smooth spatial attenuation typically associated with vignetting. Polynomial fitting therefore plays an important refinement role by improving the continuity and physical plausibility of the recovered field.

The polynomial-only method, on the other hand, lacks an explicit shared-structure constraint and is therefore more sensitive to scene-dependent radiometric variations and local image content. As a result, smooth fitting alone may not reliably separate the vignette component from non-shared radiometric variations. This explains its significantly larger errors in the experiments. In contrast, the full method combines shared low-rank modeling with low-frequency polynomial fitting, thereby leveraging both shared-component extraction and smooth-field modeling to achieve higher correction accuracy.

The current framework relies on the assumption that the vignette field remains approximately stable within a short time period under similar imaging conditions. When this assumption is satisfied, multiple images can jointly constrain a common vignette field and improve estimation stability. However, if the vignette distribution changes significantly over time or across acquisition conditions, the shared low-rank model may no longer correspond to a single stable field, and the recovered result may become a compromise estimate among multiple vignette patterns. In addition, the present formulation is based on a relatively simple low-rank structure and a smooth polynomial model, which may be insufficient for more complex cases involving adaptive rank changes or locally varying high-frequency radiometric nonuniformities.

For star images, the correction effect is further analyzed from two perspectives: the suppression of image-plane nonuniformity and the preservation of net stellar signals. As shown in Table 3 and Figure 7, the image-plane nonuniformity of the four stellar samples is clearly reduced after correction, decreasing from 1.39%, 1.82%, 1.90%, and 1.92% to 0.59%, 0.70%, 0.77%, and 0.80%, respectively. This indicates that response consistency at different field positions is significantly improved, demonstrating that the proposed method effectively suppresses the spatial radiometric nonuniformity caused by vignetting.

At the same time, the background-subtracted net stellar DN values remain largely stable, changing from 2935, 2027, 2907, and 2639 to 2932, 2027, 2881, and 2625, respectively. Compared with the pronounced improvement in the nonuniformity metric, the net DN values change only slightly, indicating that the correction mainly improves response consistency without introducing obvious bias into the intrinsic stellar radiometric signal. The visual comparison in Figure 7 also shows that the background distribution becomes more uniform after correction and the local radiometric response around the stellar targets becomes more stable. This further indicates that the proposed method effectively reduces image-plane nonuniformity while preserving net stellar radiometric information, which is beneficial for subsequent star-based radiometric calibration analysis.

Overall, the results show that combining shared low-rank modeling with polynomial fitting is an effective vignette-correction strategy. Low-rank modeling is used to identify the common vignette-related component, whereas polynomial fitting further improves its smoothness and stability, leading to better correction performance than either component alone and providing an effective solution for remote sensing image vignette correction.

## 5. Conclusions

This paper proposes a vignette correction method for remote sensing images based on low-rank modeling and polynomial fitting. By exploiting the shared vignette field among multiple images, the method constructs a data matrix in the logarithmic domain, extracts the common vignette component through low-rank decomposition, and further recovers a smooth vignette field using polynomial fitting. The final correction is performed in the original intensity domain. Overall, the proposed method provides a practical solution that balances common vignette-field extraction and smooth-field modeling.

The current method is mainly based on the assumption that the shared vignette field can be described by a low-rank model. Under more complex imaging conditions, however, the vignette field may be difficult to characterize using a fixed rank, especially a structure close to rank 1. Future work will therefore focus on adaptive rank selection or variable-rank modeling to enhance the expressive power of the model and improve robustness and generalization under more challenging scenarios.

## Figures and Tables

**Table 1:** Main notations used in the proposed algorithm.

Symbol	Description
$X=\left\{I_{k}\left(x,y\right)|k=1,\ldots N\right\}$	Input multi-frame vignetted image set
N	Number of input frames
H,W,M	Image height, width and number of pixels
$D\in R^{M\times N}$	Logarithmic data matrix constructed from vectorized images
L	Shared low-rank component
S	Sparse residual component mainly containing scene-dependent structures and local deviations
$v\in R^{M}$	Shared vignette-related vector extracted from L
$B_{0}\left(x,y\right)$	Initial vignette field reshaped from v
$\hat{B}\left(x,y\right)$	Smoothed low-frequency vignette field obtained by polynomial fitting
$\hat{V}\left(x,y\right)$	Estimated multiplicative vignette field in the intensity domain
G(x,y)	Correction gain, defined as the reciprocal of $\hat{V}\left(x,y\right)$
$I_{corr}\left(x,y\right)$	Corrected output image
P	Polynomial order
$a_{ij}$	Polynomial fitting coefficient
ϵ	Small positive constant used to avoid numerical instability

**Table 2:** Representative stars IDs and related information.

No.	RA	Dec.	Magnitude
1	63.647983	22.351181	7.846
2	108.630375	13.860219	9.153
3	124.844050	54.086008	8.284
4	161.846512	28.398869	8.683

**Table 3:** Parameters of partial-gradient and radial-polynomial methods.

Parameter	Partial-Gradient	Radial-Polynomial
Polynomial order	6	4
Gaussian standard deviation	8 pixels	80 pixels
Sampling stride	4 pixels	4 pixels
Robust iterations	5	5
Huber parameter	1.345	1.345
Edge-weight scale	2.5	2.5
Minimum weight	$10^{-3}$	$10^{-3}$
Ridge regularization	$10^{-8}$	$10^{-8}$
Center-search range	Fixed image center	±0.08 normalized coordinates
Center-search grid	/	3×3
Ellipticity range	/	0.85–1.15
Rotation candidates	/	$-15^{\circ },0^{\circ },15^{\circ }$
Central normalization radius	51 pixels	51 pixels
Correction-gain range	0.7–1.8	0.7–1.8

**Table 4:** Quantitative comparison across the four base datasets.

Method	Metric	Dataset 1	Dataset 2	Dataset 3	Dataset 4	Mean ± SD
Full	MAE	0.301	0.433	0.471	0.721	0.482 ± 0.176
	MAD	2.349	3.039	3.826	5.373	3.647 ± 1.299
	Center-MAE	0.136	0.146	0.120	0.149	0.138 ± 0.013
	Edge-MAE	0.286	0.410	0.527	0.853	0.519 ± 0.243
Low-rank only	MAE	0.243	0.423	0.580	1.254	0.625 ± 0.441
	MAD	2.986	3.316	3.193	12.233	5.432 ± 4.536
	Center-MAE	0.234	0.194	0.523	0.993	0.486 ± 0.368
	Edge-MAE	0.244	0.426	0.610	1.313	0.648 ± 0.468
Polynomial only	MAE	1.420	2.739	1.294	13.621	4.768 ± 5.938
	MAD	4.674	13.203	8.074	73.621	24.893 ± 32.674
	Center-MAE	0.436	0.504	0.491	8.799	2.558 ± 4.161
	Edge-MAE	1.596	3.103	1.324	14.469	5.123 ± 6.280

**Table 5:** Comparison of the proposed rank-1 model and standard RPCA using all available frames.

Method	Statistic	MAE (%)	MAD (%)	Center-MAE (%)	Edge-MAE (%)
Ours	Mean	0.48	3.65	0.13	0.51
	SD	0.17	1.59	0.07	0.21
	95% CI half-width	0.07	0.62	0.03	0.08
Standard RPCA	Mean	0.46	3.46	0.11	0.50
	SD	0.24	1.85	0.07	0.35
	95% CI half-width	0.09	0.72	0.03	0.13

**Table 6:** Quantitative Assessment of Star Image Processing.

No.	Star Net DN Before Correction	Image Non-Uniformity Before Correction (%)	Star Net DN After Correction	Image Non-Uniformity After Correction (%)
1	2935	1.39	2932	0.59
2	2027	1.82	2027	0.70
3	2907	1.90	2881	0.77
4	2639	1.92	2625	0.80

**Table 7:** Computational cost.

Item	Result
Image size	2048×2048
Frames used for model estimation	100
Model-estimation time	128.6±3.36 s
Mean correction time per image	0.087±0.003 s
Number of iterations	49.4±0.7
Representative convergence iterations	47
Core matrix memory	Approximately 21.9 GB
Estimated peak memory	Approximately 22–30 GB

**Table 8:** ALM convergence statistics across all evaluated datasets.

Dataset (Number of Sequences)	Iterations (Mean ± SD)	Final Normalized Residual ($\times 10^{-6}$)	Final Relative Change ($\times 10^{-5}$)
DATA-0 (7)	49.00 ± 0.00	8.545 ± 0.224	1.331 ± 0.028
DATA-1 (7)	49.71 ± 0.49	8.765 ± 0.664	1.206 ± 0.123
DATA-2 (7)	49.14 ± 0.38	9.130 ± 0.653	1.440 ± 0.118
DATA-3 (7)	49.00 ± 0.00	8.489 ± 0.076	2.013 ± 0.104
Overall (28)	49.21 ± 0.42	8.732 ± 0.520	1.497 ± 0.329

The stopping tolerances for the normalized residual and relative shared-vector change were $10^{-5}$ and $10^{-4}$, respectively. None of the 28 runs reached the maximum iteration limit of 200.

**Table 9:** Sensitivity analysis of frames $N_{f}$.

$N_{f}$	MAE (%)	MAD (%)	Center-MAE (%)	Edge-MAE (%)
5	2.87	10.57	1.07	3.25
10	1.06	6.48	0.37	1.23
20	1.63	6.72	0.49	1.89
50	0.64	4.43	0.19	0.69
100	0.52	3.95	0.14	0.56
All (depending on the dataset)	0.48	3.65	0.13	0.51

**Table 10:** Sensitivity analysis of polynomial order P.

P	Q	MAE (%)	MAD (%)	Center-MAE (%)	Edge-MAE (%)	Runtime (s)
2	6	1.37	13.48	0.14	1.80	290
4	15	0.72	7.08	0.22	0.78	270
6	28	0.42	3.72	0.10	0.50	270
8	45	0.54	3.20	0.21	0.51	274
10	66	0.45	3.40	0.19	0.50	278

**Table 11:** Sensitivity to the Sparse Regularization Parameter λ.

η	λ	MAE (%)	MAD(%)	Center-MAE (%)	Edge-MAE (%)
0.25	0.00012	0.22	2.31	0.08	0.24
0.5	0.00024	0.22	2.31	0.08	0.24
1	0.00049	0.22	2.25	0.07	0.24
2	0.00098	0.22	2.31	0.08	0.24
4	0.00195	0.22	2.31	0.08	0.24

**Table 12:** Sensitivity analysis of selected rank.

Rank	MAE (%)	MAD (%)	Center-MAE (%)	Edge-MAE (%)
1	0.20	1.14	0.07	0.15
2	0.27	1.31	0.09	0.27
3	0.27	1.34	0.09	0.27
5	0.29	1.57	0.10	0.30

**Table 13:** Correction errors obtained using joint and windowed estimation.

Estimation Strategy	Data-A MAE (%)	Data-B MAE (%)
Joint rank-1 estimation	0.68	0.40
Windowed rank-1 estimation	0.32	0.22

## Data Availability

The public datasets used in this study, including SDGSAT-1 (https://www.sdgsat.ac.cn/ (accessed on 1 May 2025), Landsat 8/9, and ASTER data (https://urs.earthdata.nasa.gov/ (accessed on 1 April 2026), are available for download from their respective websites. Due to institutional and privacy restrictions, the private datasets are not publicly available at this time; however, they may be obtained from the corresponding author upon reasonable request and with permission from the relevant institutions.

## Abbreviations

MAE	Mean Absolute Error
MAD	Maximum Absolute Deviation
Center-MAE	Center-region Mean Absolute Error
Edge-MAE	Edge-region Mean Absolute Error
RA	Right Ascension
Dec.	Declination

## References

[1] Goldman D.B. (2010). Vignette and Exposure Calibration and Compensation. IEEE Trans. Pattern Anal. Mach. Intell..

[2] Kim S.J., Pollefeys M. (2008). Robust Radiometric Calibration and Vignetting Correction. IEEE Trans. Pattern Anal. Mach. Intell..

[3] Chen C., Pan J., Wang M., Zhu Y. (2018). Side-Slither Data-Based Vignetting Correction of High-Resolution Spaceborne Camera with Optical Focal Plane Assembly. Sensors.

[4] Fan L., Yu S., Zhong X., Chen M., Wang D., Cao J., Cai X. (2023). A General Relative Radiometric Correction Method for Vignetting Noise Drift. Remote Sens..

[5] Li H., Wang X., Shen H., Yuan Q., Zhang L. (2016). An Efficient Multi-Resolution Variational Retinex Scheme for the Radiometric Correction of Airborne Remote Sensing Images. Int. J. Remote Sens..

[6] Pesta F., Bhatta S., Helder D., Mishra N. (2015). Radiometric Non-Uniformity Characterization and Correction of Landsat 8 OLI Using Earth Imagery-Based Techniques. Remote Sens..

[7] Bal A., Palus H. (2021). A Smooth Non-Iterative Local Polynomial (SNILP) Model of Image Vignetting. Sensors.

[8] Bal A., Palus H. (2023). Image Vignetting Correction Using a Deformable Radial Polynomial Model. Sensors.

[9] Shin J.-I., Cho Y.-M., Lim P.-C., Lee H.-M., Ahn H.-Y., Park C.-W., Kim T. (2020). Relative Radiometric Calibration Using Tie Points and Optimal Path Selection for UAV Images. Remote Sens..

[10] Hessel C., Grompone von Gioi R., Morel J.M., Facciolo G., Arias P., de Franchis C. (2020). Relative Radiometric Normalization Using Several Automatically Chosen Reference Images for Multi-Sensor, Multi-Temporal Series. ISPRS Ann. Photogramm. Remote Sens. Spat. Inf. Sci..

[11] Xia M., Yao J., Li L., Xie R., Liu Y. (2016). Consistent Tonal Correction for Multi-View Remote Sensing Image Mosaicking. ISPRS Ann. Photogramm. Remote Sens. Spat. Inf. Sci..

[12] Zheng Y., Lin S., Kambhamettu C., Yu J., Kang S.B. (2009). Single-Image Vignetting Correction. IEEE Trans. Pattern Anal. Mach. Intell..

[13] Yu W. (2004). Practical Anti-Vignetting Methods for Digital Cameras. IEEE Trans. Consum. Electron..

[14] Kordecki A., Palus H., Bal A. (2016). Practical Vignetting Correction Method for Digital Camera with Measurement of Surface Luminance Distribution. SIViP.

[15] Zhou T., Tao D. (2011). GoDec: Randomized Low-Rank & Sparse Matrix Decomposition in Noisy Case. Proceedings of the 28th International Conference on Machine Learning, ICML 2011.

[16] Guo K., Liu L., Xu X., Xu D., Tao D. (2018). GoDec+: Fast and Robust Low-Rank Matrix Decomposition Based on Maximum Correntropy. IEEE Trans. Neural Netw. Learn. Syst..

[17] Vaswani N., Bouwmans T., Javed S., Narayanamurthy P. (2018). Robust Subspace Learning: Robust PCA, Robust Subspace Tracking, and Robust Subspace Recovery. IEEE Signal Process. Mag..

[18] Chen Z., Wang B. (2017). Spectrally-Spatially Regularized Low-Rank and Sparse Decomposition: A Novel Method for Change Detection in Multitemporal Hyperspectral Images. Remote Sens..

[19] Yang Y., Zhang J., Liu D., Wu X. (2019). Low-Rank and Sparse Matrix Decomposition with Background Position Estimation for Hyperspectral Anomaly Detection. Infrared Phys. Technol..

[20] Roy S., Carass A., Prince J.L. (2011). Compressed Sensing Based Intensity Non-Uniformity Correction. Proceedings of the 2011 IEEE International Symposium on Biomedical Imaging: From Nano to Macro.

[21] Meygret A., Blanchet G., Latry C., Kelbert A., Gross-Colzy L. (2019). On-Orbit Star-Based Calibration and Modulation Transfer Function Measurements for PLEIADES High-Resolution Optical Sensors. IEEE Trans. Geosci. Remote Sens..

[22] Xu C. (2017). A Flux Calibration Method for Remote Sensing Satellites Using Stars. arXiv.

[23] Howell S.B. (1989). Two-Dimensional Aperture Photometry Signal-to-Noise Ratio of Point-Source Observations and Optimal Data-Extraction Techniques. Publ. Astron. Soc. Pac..

[24] Newberry M.V. (1991). Signal-to-Noise Considerations for Sky-Subtracted Ccd Data. Publ. Astron. Soc. Pac..

[25] Li B., Ren W., Fu D., Tao D., Feng D., Zeng W., Wang Z. (2019). Benchmarking Single-Image Dehazing and Beyond. IEEE Trans. Image Process..

[26] Sakaridis C., Dai D., Van Gool L. (2018). Semantic Foggy Scene Understanding with Synthetic Data. Int. J. Comput. Vis..

[27] Cai B., Xu X., Jia K., Qing C., Tao D. (2016). DehazeNet: An End-to-End System for Single Image Haze Removal. IEEE Trans. Image Process..

[28] Li B., Peng X., Wang Z., Xu J., Feng D. (2017). AOD-Net: All-in-One Dehazing Network. Proceedings of the 2017 IEEE International Conference on Computer Vision (ICCV).

[29] Candès E.J., Li X., Ma Y., Wright J. (2011). Robust Principal Component Analysis?. J. ACM.

[30] Gao H., Cai J.-F., Shen Z., Zhao H. (2011). Robust Principal Component Analysis-Based Four-Dimensional Computed Tomography. Phys. Med. Biol..

